# COVID-19: A Syndemic Requiring an Integrated Approach for Marginalized Populations

**DOI:** 10.3389/fpubh.2021.675280

**Published:** 2021-05-11

**Authors:** Rosemary M. Caron, Amanda Rodrigues Amorim Adegboye

**Affiliations:** ^1^Department of Health Management and Policy, Master of Public Health Program, College of Health and Human Services, University of New Hampshire, Durham, NH, United States; ^2^School of Nursing, Midwifery, and Health, Faculty of Health and Life Sciences, Coventry University, Coventry, United Kingdom

**Keywords:** COVID-19, health inequities, ethnic minorities, immigration, refugees, syndemic, essential public health services

## Abstract

The novel coronavirus, SARS-CoV-2, responsible for the COVID-19 pandemic, has challenged healthcare systems globally. The health inequities experienced by immigrants, refugees, and racial/ethnic minorities have been aggravated during the COVID-19 pandemic. The socioeconomic, political, and demographic profile of these vulnerable populations places them at increased risk of contracting COVID-19 and experiencing significant morbidity and mortality. Thus, the burden of the COVID-19 pandemic is disproportionally higher among these at-risk groups. The purpose of this perspective is to: (1) highlight the interactions among the social determinants of health (SDoH) and their bi-directional relationship with the COVID-19 pandemic which results in the current syndemic and; (2) offer recommendations that consider an integrated approach to mitigate COVID-19 risk for marginalized populations in general. For these at-risk populations, we discuss how individual, structural, sociocultural, and socioeconomic factors interact with each other to result in a disparate risk to contracting and transmitting COVID-19. Marginalized populations are the world's collective responsibility. We recommend implementing the Essential Public Health Services (EPHS) framework to promote those systems and policies that enable optimal health for all while removing systemic and structural barriers that have created health inequities. The pledge of “Health for All” is often well-accepted in theory, but the intricacy of its practical execution is not sufficiently recognized during this COVID-19 syndemic and beyond.

“*Where there is life, there is hope.”*

-R.I. Kelly, Irish Immigrant.

## Introduction

A novel coronavirus, SARS-CoV-2, responsible for the current Coronavirus Disease 2019 (COVID-19) pandemic, has left no population sector untouched. At the time of this writing, there are 133,710,116 confirmed cases and 2,899,031 deaths worldwide (1). High- and middle-income countries had the highest incidence and mortality rates per million population in the early months of the COVID-19 pandemic (2). Despite differences in the public health management of the pandemic across countries (e.g., testing capacity and timing of testing, quarantine order implementation, availability of healthcare services) and due to the natural progression of the virus, ~215 countries and territories worldwide have experienced the health and economic effects of the COVID-19 pandemic to date (3, 4).

Further, the COVID-19 burden is unevenly distributed within countries with vulnerable populations who are at a higher risk for presenting with more severe illness and dying from the disease (5). Vulnerable populations that are marginalized include, but are not limited to, the elderly, children, disabled, underinsured, socioeconomically disadvantaged, incarcerated, abused and the mentally ill (6). Immigrants, refugees, and racial/ethnic minorities also represent marginalized groups and will comprise the populations of focus herein.^1^ Based on the unique and overlapping challenges many, but not all, immigrants, refugees, and racial/ethnic minorities encounter during their settlement and way of life (e.g., unfamiliarity with a host country's language, are at different stages of acculturation, possess an inadequate health literacy level, experience unemployment, poor housing quality, and food insecurity), these groups have experienced variable susceptibility to COVID-19 morbidity and mortality during this dynamic pandemic (6, 7). These types of representative inequities are interrelated and exacerbate the effects of COVID-19, and vice versa, on marginalized populations to varying extents (5, 6, 8, 9). For example, during the COVID-19 pandemic in the United Kingdom (UK), the all-cause mortality rate was higher at times for migrants than those born in the UK (10).

The World Health Organization (WHO) declared COVID-19 a pandemic in March 2020 that resulted in the implementation of control measures, such as the closure of geographic borders and lockdown policies which further impacted the risks and drivers of migration by decreasing movement for populations and posing challenges for managing (e.g., testing, contact tracing, treatment) the pandemic (7). For example, refugee resettlement has significantly slowed with “…168 countries fully or partially closing their boundaries at the height of the crisis. Of these 168 countries, ~90 made no exception for those seeking asylum, and some have pushed asylum seekers back to their countries of origin” (11). Additionally, some governments have unfairly blamed immigrants for the COVID-19 pandemic and this has resulted in widespread xenophobia and anti-immigration policies (12).

COVID-19 prevention efforts have primarily taken the form of social distancing, mask-wearing, hand hygiene, disinfection of surfaces and staying at home when one is sick (8). Until there is a widely available and effective COVID-19 vaccine that has been extensively administered, it is estimated that the global economic impact from the pandemic could be $3.4 trillion per year. This would translate to a cost of ~$983 billion (5.6% of GDP) for the European Union (EU); $145 billion (4.3% of GDP) for the UK, and; $480 billion (2.2% of GDP) for the United States of America (USA) (13). Further, it is estimated that the USA, the UK, the EU, and other high-income countries together could lose ~$119 billion a year if the poorest countries are denied a vaccine supply (13).

To date, the impact of the COVID-19 pandemic has been widespread affecting social systems, such as our healthcare, public health, employment, education, housing, and food at all levels. The WHO (14) predicts that the resultant economic and social disruption could significantly increase, by an order of ten million to a hundred million, the number of people at risk for falling into extreme poverty and malnutrition. The effects on the essential services provided by these systems are further compounded by the closure of countries' borders, restrictions on trade, and quarantine and isolation measures that all converge to significantly impact marginalized populations, especially immigrants, refugees, and racial/ethnic minorities (14).

As the world wrestles a pandemic that forces millions of people into confinement and social distancing, asylum seekers, and refugees continue to be stranded in camps and informal settlements. The WHO (7) surveyed 30,000 refugees and migrants globally to determine the impact COVID-19 has had on their lives. More than half reported that COVID-19 negatively impacted their mental health status, led to increased substance use, and overall worsened their quality of life which prior to the pandemic already involved harsh living conditions, including a lack of access to healthcare services, housing, water, and sanitation, for many. Ethnic minorities and immigrants are significantly affected groups, mainly due to intersecting inequities based on their migration status, ethnicity/race, and socioeconomic position which corroborate unequal access to quality health care (15). For example, immigrant populations are reported to experience a higher COVID-19 risk compared to the native-born of the country in which they reside due to a higher incidence of poverty, overcrowded housing, and unstable employment for which it is challenging to practice social distancing guidelines. The children of immigrants also experience a disadvantage in that their parents have fewer resources to help home-school them and 40% of children of immigrant parents do not speak the host country's language (10, 16). Additionally, a higher burden of COVID-19 cases and deaths has been reported among racial/ethnic minorities who live in poverty, overcrowded housing, and experience racial and economic segregation (9). Navigating the COVID-19 pandemic is further compounded for these marginalized populations due to varying degrees of fear, financial constraints, cultural and language barriers and exclusion from health education and treatment programs (12). Lastly, these population groups also generally have poor access to public health and medical care and higher rates of chronic diseases that place them at elevated risk for contracting COVID-19 and experiencing severe complications (17). In sum, COVID-19 has disproportionately affected vulnerable populations globally due to the existence of inequities and limited mitigation capacity that is compounded by comorbidities, and insufficient public health and healthcare access, prevention, and treatment options (17).

The purpose of this perspective is to: (1) highlight the interactions among the social determinants of health (SDoH) and their bi-directional relationship with the COVID-19 pandemic which results in the current syndemic and; (2) offer recommendations that consider an integrated approach to mitigate COVID-19 risk for marginalized populations in general.

## COVID-19 as a Syndemic

Singer (18) developed the concept of a syndemic in the 1990s when studying the interconnection among HIV/AIDS cases, violence, and substance use. A syndemic approach investigates the reasons why certain diseases cluster and affect specific individuals and groups; the pathways through which they interact biologically, and thus impact their overall disease burden; while considering how the social environment (e.g., inequality, inequity, and disparity) contributes to disease clustering, interaction, and vulnerability (19). A syndemic, also referred to as a synergistic pandemic, describes the interactions among health determinants that can contribute to an individual's susceptibility to disease, therefore potentially promoting the establishment of an epidemic or pandemic (20). Bambra et al. (21) further describe that a syndemic occurs when risk factors or comorbidities are interconnected and cumulative, thus adversely exacerbating the pre-existing disease burden and its unfavorable effects. Accordingly, a syndemic has been simply defined as the synergistic interaction between biological and socioecological factors (21). Syndemics have been described for other multifactorial conditions as obesity; HIV, malnutrition and food insecurity in sub-Saharan Africa; and violence, immigration, depression, type 2 diabetes and abuse in women who have emigrated to the USA from Mexico (22, 23).

The SDoH, which have been estimated to account for 80–90% of one's health outcomes, represent key factors in our living environment (i.e., the places we work, play, learn, worship, and age) that contribute to our overall health status and quality of life (24, 25). Representative SDoH categories include access to healthcare services, social and community context, education, economic stability, neighborhood and the built environment (25). It has been proposed that by addressing the SDoH, the population's susceptibility to disease outbreaks could be significantly reduced (20). In brief, the syndemic approach recognizes how social realities shape individuals' or communities' illness experiences along with the distribution of diseases across populations (26). Furthermore, the SDoH are influenced by the distribution of currency, power, and resources at various governmental levels (27).

Bambra et al. (21) argue, and we agree, that for marginalized populations, COVID-19 is experienced as a syndemic that interacts with and exacerbates their pre-existing non-communicable diseases (NCDs) and social conditions. The authors state that marginalized populations, specifically, minority ethnic populations in the USA, Europe, and other high-income countries, and those living in poverty, generally experience several coexisting NCDs (e.g., obesity, diabetes, heart disease, etc.) which are also known risk factors for COVID-19 (21). The Gypsy/Roma population, for example, a marginalized minority ethnic population in Europe, is estimated to exhibit a smoking rate that is more than double the European average which places this population at an elevated risk for respiratory illnesses and COVID-19 (28). The observed health inequities primarily occur due to, or are influenced by, the SDoH (e.g., poor quality housing, unsafe working conditions, unemployment, lack of access to quality healthcare services, and lack of access to healthy food, clean water, and adequate sanitation). These SDoH can interact synergistically and amplify their deleterious effects, such as unemployment and homelessness, or poor-quality housing and food deserts, thus affecting a population's health status and vulnerability to disease (25).

However, the risk of contracting COVID-19 among these vulnerable populations is also elevated in the absence of underlying or pre-existing health conditions. For example, studies have demonstrated that adverse psychosocial circumstances (e.g., stress, anxiety, loneliness, hopelessness) may increase the susceptibility to COVID-19 (21). Yadav et al. (29) further report that countries with higher social and economic inequalities have more people living with coexisting NCDs and therefore they are more vulnerable to the syndemic impact of COVID-19. The identified risk factors (i.e., poorly managed pre-existing health conditions, poverty, inadequate housing, stress, anxiety, etc.) for contracting COVID-19 are not new to marginalized populations. The COVID-19 syndemic has served to intensify their daily struggles and contribute to excessive morbidity and mortality.

**Figure 1:**
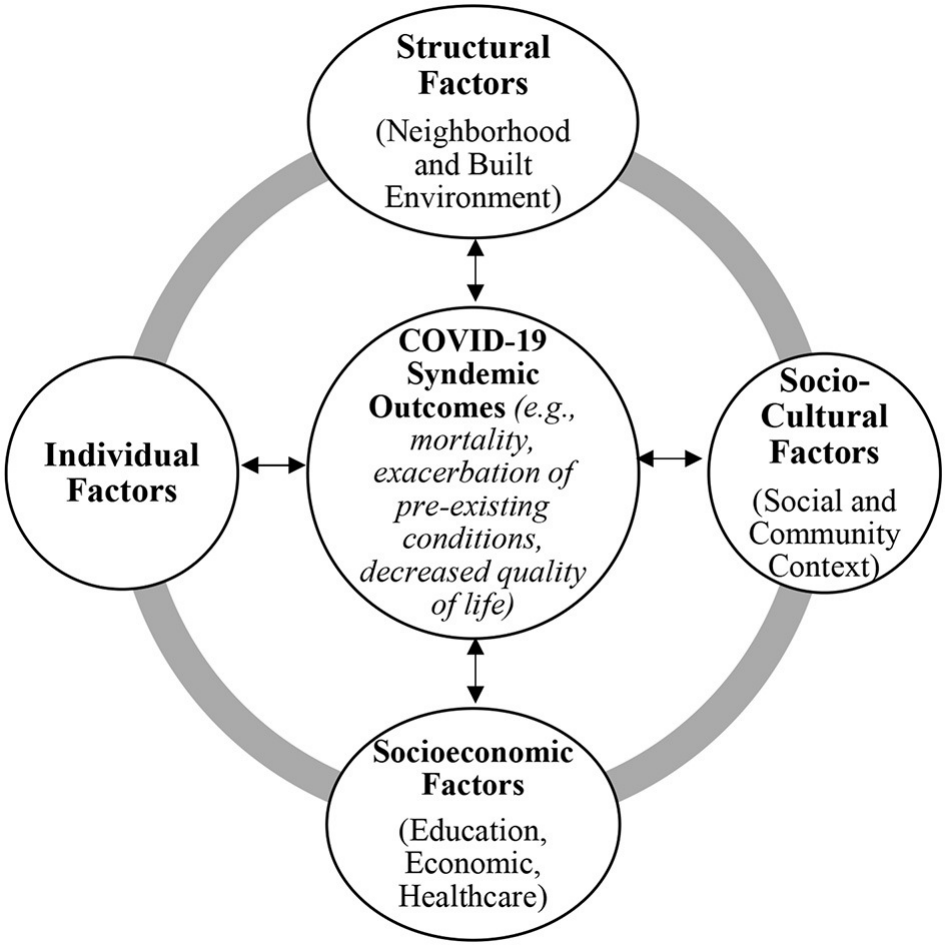
Interactions among the SDoH and the Bi-directional Relationship with the COVID-19 Syndemic. Social determinants of health (SDoH) that can interact to influence health outcomes for all populations with specific emphasis on the COVID-19 syndemic are illustrated in this figure. Concurrently, the COVID-19 syndemic will influence these health factors, as shown by the double-sided arrows. The gray outer circle illustrates the connection among the SDoH shown. The main categories illustrated are adapted from Mendenhall (26) and the model is adapted from HealthyPeople.gov Social Determinants of Health (25). Representative examples for each category are listed here: Structural Factors (e.g., poverty, food insecurity, poor quality housing, violence); Sociocultural Factors (e.g., acculturation, identity, lifestyle, family conflict and support, institutional support); Socioeconomic Factors (e.g., language and literacy, employment, access to primary care); and Individual Factors (e.g., genetics, health behavior, self-efficacy).

Worldwide COVID-19 cases to date have resulted in the identification of several risk factors including pre-existing health conditions (e.g., heart disease, chronic respiratory disease, cancer, diabetes, and obesity) in addition to adverse SDoH that leave many susceptible to the disease (21, 29, 30). Figure 1 illustrates how the SDoH co-exist with comorbidities (e.g., obesity, diabetes, cancer, etc.) and COVID-19 experienced by all populations interact to augment this syndemic. We illustrate the interactions among the SDoH and the bi-directional relationship with the COVID-19 syndemic that is important to consider when developing interventions for marginalized populations. The SDoH explain, in part, the health status of marginalized and non-marginalized populations (25). The SDoH do not discriminate and they impact all populations, however, to different extents, thus representing an important point in which to intervene to improve the population's health.

The effects of these SDoH and their resultant negative health conditions may be magnified in a syndemic. Thus, we propose that focusing political efforts to improve the SDoH may hold the potential to significantly influence the health outcomes for marginalized populations and thereby reduce the synergistic effects among a population's pre-existing health status and SDoH and create an overall healthier and/or more resilient population.

## The Burden of COVID-19 Among Marginalized Populations in High-Income Countries

Immigrants, refugees, and racial/ethnic minorities are generally a disproportionately at-risk population to infectious diseases due to overcrowded and unsanitary living conditions, inadequate access to quality health services, or total exclusion from national healthcare plans, lack of access to education and nutritious food, infrequent employment, living in poverty, and pre-existing health conditions (31). Thus, the inequitable living conditions many in these vulnerable populations experience daily varies by the host country, community of residence, and among populations themselves, thus placing them at variable risk for COVID-19 infection, transmission, morbidity, and mortality (16, 32). This is illustrated by the overcrowded camps in Greece where several COVID-19 outbreaks have been reported despite calls for relief (33). For example, it has been reported that more than 60,000 refugees and immigrants live in 36 Greek reception centers and camps, in which the majority are overcrowded and lack basic infrastructure (33). These conditions preclude early detection, testing, diagnosis, and contact tracing of COVID-19 cases, thus making it difficult to provide timely health care for refugees and immigrants suffering from COVID-19 and potentially increasing the disease transmission rate (31). Additionally, a population-based cohort study conducted in Sweden demonstrated a higher risk of mortality from COVID-19 compared to all other causes of death for the following factors: being male, having less personal income, lower education level, and being single. For immigrants from a low- or middle-income country, a higher risk of mortality from COVID-19 was predicted but not for all causes of death. The authors conclude that the interaction of SARS-CoV-2 and the social environment exerts an unequal COVID-19 burden on the most vulnerable populations (34).

Research to date has demonstrated that a significant predictor of elevated COVID-19 mortality rates at the USA county level are adverse SDoH. Specifically, COVID-19 mortality rates in the USA are elevated in counties with a higher percentage of adverse SDoH, Black residents, uninsured adults, low birth weight babies, incarceration, and those without a high school diploma and internet access (35). At the time of this writing, there have been 30,996,784 cases and 560,035 deaths attributed to COVID-19 in the USA and 4,370,321 cases and 149,968 deaths attributed to COVID-19 in the UK (1, 36). Black, Indigenous, Hispanic, and other people of color suffer a higher mortality rate by COVID-19 with Black people dying from COVID-19 at 1.5 times the rate of White people in the USA (37). The Centers for Disease Control and Prevention (CDC) estimate that all age groups in the Black population and only the working-age population among Hispanics experience higher COVID-19 mortality compared to their White counterparts in the USA (16). After controlling for demographics, socioeconomic factors, and pre-existing health conditions, minorities in the USA have the highest mortality rate from most causes (16). Race and ethnicity data are known for 77% of deaths attributed to COVID-19 in the USA, thus the magnitude of this finding could be even more significant (38).

The Organization for Economic and Cooperation and Development (16) reports that many countries do not have statistical data on COVID-19 deaths for immigrants but do so for racial/ethnic minorities. For example, in the UK, one-third of critically ill COVID-19 patients were registered as Black Asian and Minority Ethnic (16). The OECD (16) reports that when considering socioeconomic and environmental factors, COVID-19 mortality remains elevated for Black men (2.0 times), Black women (1.4 times) and South Asian men (1.5 times) globally compared to their White counterparts. Additionally, structural racism has further contributed to the COVID-19 risk in these marginalized groups since the social systems (e.g., healthcare, education, employment, justice) in place perpetuate practices that consent or promote biased or prejudiced beliefs, stereotypes, and inequitable distribution of resources (39).

Overall, mortality data for COVID-19 among immigrants is incomplete due to missing death certificate data, but crude assessments conclude that immigrants have suffered a higher COVID-19 incidence and mortality rate (16). This finding could be due to the elevated prevalence of poverty, overcrowded and unsanitary living conditions, and the availability of employment where practicing social distancing is challenging, for example, thus leaving immigrants at a higher risk of COVID-19 infection than the native-born of the countries in which they reside (16). Similar adverse SDoH can also impact refugee populations globally. The International Rescue Committee estimates that 34 countries experiencing conflict and a fragile infrastructure could experience ~1 billion COVID-19 infections and 3.2 million deaths (11). Similar to the immigrant population, public health data regarding COVID-19 prevalence and mortality data for refugees is limited. It is important to note that the social interactions in the community contribute to a marginalized population's COVID-19 risk (e.g., time spent living in the community, familiarity and utilization of social and healthcare services, language, and acculturation proficiency, etc.) and warrant further investigation when preparing for and responding to public health events.

## Discussion

Immigrants, refugees, and racial/ethnic minorities are often forgotten during non-syndemic conditions and thus, these people may not have access to the health education and healthcare access required for protecting themselves from contracting COVID-19 not only due to health literacy and financial issues but especially now that social systems are stressed and not working in a capacity to mitigate health inequities which have been aggravated during the COVID-19 syndemic. The burden of the COVID-19 syndemic (e.g., physical, mental health, and socioeconomic consequences) is disproportionally higher among these groups (5, 16). This unequal impact warrants them to be considered as one of the most at-risk groups in public health policies, including access to health care, vaccine, and COVID-19 relief efforts. The WHO and the UN Refugee Agency have recommended that disease surveillance and national health systems should integrate refugees and immigrants, prioritize decongestion of camps, and provide safe accommodation to vulnerable people, as part of a holistic effort to respond to the COVID-19 syndemic crisis (31, 40).

There is also a misleading belief that a highly effective vaccine against the SARS-COV-2 virus is a panacea for the syndemic. Although the vaccine is the best global preventive measure, it is unlikely to eradicate the virus completely and in many high-income countries, immigrants have been alienated from the vaccine agenda, thus raising a contentious discussion on the ethical distribution of the COVID-19 vaccine (41). Furthermore, the WHO (12) estimates that ~1 billion people have been displaced or are migrating due to the COVID-19 syndemic. Efforts to control the spread of the disease are either unattainable or possess dire consequences for this population. For example, refugee camps traditionally lack space to practice social distancing and there is often an absence of clean water, thus prohibiting sanitary practices. Further, food shortages, limited employment opportunities, insufficient COVID-19 testing and treatment capacity will all contribute to a recovery delay among these vulnerable populations (11).

The Declaration of Alma-Ata was developed to promote health and prevent disease among all populations (42). This declaration focused primarily on the establishment of primary care which we contend is still quite relevant to the syndemic conditions that allow for the prevalence of COVID-19 in our communities. This fundamental document promotes an inter-sectoral and inter-disciplinary approach that fulfills a public health and primary healthcare mission with a sustainable mindset that is cognizant of the effects of the social, political, and economic determinants of health and the requisite resources to effect change. As we revisit this document, we are also drawn to the CDC's revised Essential Public Health Services (EPHS) which we propose may serve as a navigation tool that is grounded in an integrated approach for achieving the mission of improving “Health for All.”

The public health mission is to assure conditions in which people can be healthy wherever they live and work. The government of every country should have a role in fulfilling this mission by addressing the core functions of public health which include the following: assessing the health status and needs of the populations they serve; developing policy that is protective of the public's health; and assuring that the necessary services are available to create conditions in which people may attain health (43). These core public health functions are achieved *via* conducting EPHS at a local and more broad geographic level. Specifically, the assessment core function requires that a public health entity routinely and methodically collect and analyze information regarding the health needs and health status of a community (44). This activity is accomplished by two main EPHS: (1) assess and monitor the population's health status and factors that influence health, community needs and assets; and (2) investigate, diagnose, and address health problems and hazards affecting the population (43, 44).

The development of policy that is protective of the public's health includes four EPHS: (1) communicate effectively to inform and educate people about health factors and how to improve health; (2) strengthen, support, and mobilize communities and partnerships to improve health collectively; (3) create, champion, and implement policies, plans, and laws that positively impact health for populations; and (4) utilize legal and regulatory actions developed to improve and protect the public's health (43, 44).

In order to assure that the necessary services are in place to create a culture of health for populations four EPHS are required: (1) assure an effective public health system that enables equitable access to healthcare services; (2) educate and support a diverse and competent public health workforce; (3) improve public health functions through continuous quality improvement, research and evaluation; and (4) develop and maintain a robust, sustainable, and organized infrastructure for public health (43, 44).

It is important to note that assessing, addressing, and evaluating the contribution of health determinants (e.g., social, economic, political, environmental) is inherent in applying the EPHS. We propose the EPHS framework and their examples in Table 1 for consideration when attempting to achieve “Health for All.”

**Table 1:** Recommendations to improve the WHO priority of “Health for All” *via* the CDC's Essential Public Health Services (EPHS).

**Core function of public health: assessment**
1. Assess and monitor population health status, factors that influence health, and community needs and assets.
•Example: Identification of health determinants and risks and the determination of health service needs in a population (e.g., community health assessment, disease or immunization registry).
2. Investigate, diagnose, and address health problems and hazards affecting the population.
• Example: Timely identification and investigation of health threats (e.g., infectious disease, chronic disease, injury, environmental hazards).
**Core function of public health: policy development**
3. Communicate effectively to inform and educate people about health, factors that influence it, and how to improve it.
• Example: Health communication, education, information, and promotion efforts delivered in a culturally and linguistically literate manner to impacted populations.
4. Strengthen, support, and mobilize communities and partnerships to improve health.
• Example: Build coalitions, partnerships, and alliances that act to improve community health (e.g., housing authority, law enforcement, schools, community organizations).
5. Create, champion, and implement policies, plans, and laws that impact health.
• Example: Development and enactment of policy, codes, regulations, and legislation to protect the population's health (e.g., safe and affordable housing that reduces homelessness).
6. Utilize legal and regulatory actions designed to improve and protect the public's health.
• Example: Encourage compliance with public health regulations *via* enforcement approaches (e.g., fines for code violations).
**Core function of public health: assurance**
7. Assure an effective system that enables equitable access to the individual services and care needed to be healthy.
• Example: Assuring the identification and linkage of people to appropriate and coordinated health care (e.g., community health clinic, hospital, specialty care).
8. Build and support a diverse and skilled public health workforce.
• Example: Implementation of life-long learning for public health professionals.
9. Improve and innovate public health functions through ongoing evaluation, research, and continuous quality improvement.
• Example: Critical review of health program utilization and effectiveness.
10. Build and maintain a strong organizational infrastructure for public health.
• Example: Implement policy based on evidence-based research.

*Sources: CDC (44, 45)*.

These core functions of public health are achieved by systematically accomplishing the aforementioned EPHS for a population. The foundation of these services is grounded in a governmental responsibility to see that they are enacted. For example, in the USA, the individual states are encouraged to lead the charge for conducting the public health mission. The federal level of government is responsible for establishing national health objectives and priorities, providing technical assistance to states, and funding activities aimed at improving the population's health (43). We propose that regardless of the functionality of a country's governmental public health infrastructure, an effort should be made, in conjunction with, or by, non-governmental and/or community health organizations to implement select EPHS that are achievable. If it is not possible to enact the EPHS comprehensively, then we recommend that communities, based on their available resources, select those that will help make the most progress in improving the health of their respective populations. Selecting an EPHS from each core function would be a reasonable approach to help prioritize the public health issues for a community. EPHS that could be implemented by a governmental or non-governmental or community entity to mitigate the COVID-19 syndemic effects on marginalized populations include the following:

- Routinely collect COVID-19 prevalence, mortality, risk factor, and SDoH information *via* a door-to-door survey to help assess the extent and severity of the disease spread.
- Establishing or maintaining a disease registry will help with epidemiological investigations, including the allocation of scarce resources to areas of a community experiencing significant disease outbreaks.
- For COVID-19 outbreaks in a community, the provision of contact tracing would help to address the potential for the spread of the disease in the community.
- COVID-19 health education, including signs and symptoms, testing and treatment locations, and transmission prevention should be communicated in a manner and language that is most effective for comprehension by the target audience (e.g., community meetings, public service announcements on the television, radio, print, or social media platforms).
- Establish community coalitions comprised of multi-sector representatives (e.g., housing, school, law enforcement, healthcare and public health organization, transportation, housing, etc.) including inviting members from the at-risk population to have a voice in the development of COVID-19 mitigation approaches that are acceptable to the community.
- Use community-based partnerships to advocate for freely available COVID-19 testing and vaccination sites for which demographic and COVID-19 status information is collected.
- Encourage compliance with public health management approaches including COVID-19 testing, quarantine and isolation orders, and acquiring an available, effective vaccine to assist in mitigation efforts.
- Integrate healthcare, social, and economic services at community clinics (e.g., COVID-19 testing and vaccination services, foodbank, and employment opportunities in one common location). Awareness of the SDoH that adversely impact the population's health in a community is important to know to provide resources to combat these potential contributors to negative health outcomes.
- Encourage the entry into and continuation of lifelong learning of the public health workforce so appropriately trained personnel will have sufficient knowledge and skills to assist, in a culturally tailored approach, vulnerable populations in light of the increased risks they experience.
- Continuously evaluate mobile and stationary COVID-19 testing and vaccination sites that serve these communities. Identify the barriers that inhibit participation in disease prevention efforts. Emphasize their benefit and remove any associated stigma by working with the community.
- Establish a public health infrastructure that considers the population's challenges and acknowledges barriers that have been overcome. The infrastructure must start somewhere so any entity that takes action to promote health, prevent disease, and protect the health of vulnerable populations is a step that helps to protect the “Health for All.”

The EPHS framework promotes those systems and policies that enable optimal “Health for All” while removing systemic and structural barriers (e.g., poverty, discrimination, racism) that have created health inequities (44). We acknowledge the existing barriers that impact the achievement of adequate health status for marginalized populations, yet we encourage governments and organizations to consider the EPHS framework and select a starting point to address so that over time, this framework will become an accepted and sustained approach to improve “Health for All.” The EPHS framework has the potential to show the strengths and weaknesses in a government's public health infrastructure and thus, is a valuable tool to prioritize and allocate scarce resources to protect the population. We have a long way to go to achieve this ambitious goal but if countries could collaborate further *via* the EPHS framework to address the SDoH that favor negative health outcomes then there is hope that we will prevail over the current COVID-19 syndemic and be well on our way to be prepared for the next global public health challenge.

## Data Availability Statement

The original contributions presented in the study are included in the article/supplementary material, further inquiries can be directed to the corresponding author.

## Author Contributions

RC proposed the topic for discussion. RC and AA co-developed the manuscript outline and co-wrote the article. All authors contributed to the article and approved the submitted version.

## Conflict of Interest

The authors declare that the research was conducted in the absence of any commercial or financial relationships that could be construed as a potential conflict of interest.

## Footnotes

^1^The terms “migrants”, “immigrants”, and “foreign-born” are used synonymously. These terms reference all persons born abroad, regardless of their migration category, legal status, or nationality. The term “refugee” is used to describe “someone who has been forced to flee his or her country because of persecution, war or violence” per the United Nations High Commissioner for Refugees definition (available at https://www.unrefugees.org/refugee-facts/what-is-a-refugee/.) “Racial/Ethnic minority” is defined as “a group of people of a particular race or nationality living in a country or area where most people are from a different race or nationality (available at https://dictionary.cambridge.org/us/dictionary/english/ethnic-minority).
